# First Principles Study on the CO Oxidation on Mn-Embedded Divacancy Graphene

**DOI:** 10.3389/fchem.2018.00187

**Published:** 2018-05-29

**Authors:** Quanguo Jiang, Jianfeng Zhang, Zhimin Ao, Huajie Huang, Haiyan He, Yuping Wu

**Affiliations:** ^1^College of Mechanics and Materials, Hohai University, Nanjing, China; ^2^Guangzhou Key Laboratory of Environmental Catalysis and Pollution Control, Institute of Environmental Health and Pollution Control, School of Environmental Science and Engineering, Guangdong University of Technology, Guangzhou, China

**Keywords:** graphene, divacancy, Mn-embedded, CO oxidation, first principles calculations

## Abstract

The CO oxidation mechanism on graphene with divacancy (DG) embedded with transition metal from Sc to Zn has been studied by using first principles calculations. The results indicate that O_2_ molecule is preferentially adsorbed on Sc, Ti, V, Cr, Mn, and Fe-DG, which can avoid the CO poisoning problem that many catalysts facing and is beneficial to the CO oxidation progress. Further study indicates that Mn-DG shows the best catalytic properties for CO oxidation with consideration of both Langmuir-Hinshelwood (LH) and Eley-Rideal (ER) oxidation mechanisms. Along the ER mechanism, the reaction energy barrier for the first step (CO _free_ + O_2__pre-adsorbed_ → OOCO) is 0.96 eV. Along the LH mechanism, the energy barrier for the rate limiting step (CO _adsorbed_ + O_2__adsorbed_ → OOCO) is only 0.41 eV, indicating that the CO oxidation on Mn-DG will occur along LH mechanism. The Hirshfeld charge distributions of O_2_ and CO molecules is tuned by the embedded Mn atom, and the charge transfer from the embedded Mn atom to the adsorbed molecules plays an important role for the CO oxidation. The result shows that the Mn-embedded divacancy graphene is a noble-metal free and efficient catalyst for CO oxidation at low temperature.

## Introduction

Carbon monoxide (CO) is colorless, tasteless and toxic in air, while oxidation of CO is an efficient way to eliminate of the air pollutant (Xie et al., 2009). CO oxidation has important applications in atmosphere purification for hydrogen gas fuel in fuel cells as well (Qiao et al., 2015; Saavedra et al., 2016). Noble metals(Bleakley and Hu, 1999; Zhang and Hu, 2001; Liu et al., 2002; Gong et al., 2004; Dupont et al., 2006; Zhang et al., 2006; Liu, 2007) are common catalysts for the CO oxidation, where the rate limiting energy barriers are 0.46 eV (Liu et al., 2002) for Au(221), 0.91 eV (Gong et al., 2004) and 0.93 eV (Zhang and Hu, 2001) for Pd(111), 0.79 eV (Gong et al., 2004) and 0.82 eV (Dupont et al., 2006) for Pt(111), 1.17 eV (Gong et al., 2004) and 1.01 eV (Zhang et al., 2006) for Rh(111), 1.00 eV (Liu, 2007) for Rh(100). Due to the high cost and high reaction temperature of these noble metals, it is desirable to develop noble-metal-free catalysts for CO oxidation at low temperature. Noble metal clusters on supports are further studied to decrease the reaction barriers for CO oxidation (Tang et al., 2015; Ma et al., 2016; Wang et al., 2016; Ali et al., 2017; Chen et al., 2017). Furthermore, single atom catalyst decorated on appropriate matrix is attracted a lot of interests due to the excellent catalytic performance (Dvorák et al., 2016; Jones et al., 2016). The outstanding physical properties (Novoselov et al., 2005; Balandin et al., 2008; Lee et al., 2008) and the large surface-to-volume ratio make graphene (Novoselov et al., 2004) to be a promising substrate to realize high performance single atom catalysis. However, the inert nature of pristine graphene usually causes clustering problems for the adsorbed metal atoms (Liu and Huang, 2017; Liu et al., 2017). The interactions between the pristine graphene and the adsorbed atoms can be enhanced by introducing different carbon vacancies. Experimentally, single carbon vacancy (SV) and double carbon vacancies (DV) are common point defects on graphene. Vacancies can be introduced into graphene by exposing it to the focussed electron beam, and the vacancy defects in graphene can be tailored by controlling the exposing time, where the DV is more often observed than SV due to the high energy of the SV under electron beam irradiation (Robertson et al., 2012). In addition, the concentration of DV changes from 0.1 to 0.5 nm^2^ by controlling the total electron beam dose (Robertson et al., 2012). In addition, it is reported that the metal atoms resident on the SV and DV positions in graphene surfaces are stable in comparison to that on graphene edge (Robertson et al., 2013). Hence, it is important to evaluate the catalytic performance of a single atom supported on graphene with both SV and DV defects.

Many works about the CO oxidation have been reported for single atom decorated at the single carbon vacancy on graphene. Theoretically, Au- (Lu et al., 2009), Fe- (Li et al., 2010), Cu- (Song et al., 2011), Pt- (Tang et al., 2012), Si- (Zhao et al., 2012), and Al-embedded (Jiang et al., 2014) SV graphenes show high activity for the CO oxidation. Although the SV graphene with decorating metal atoms has high catalytic activity, decorating metal atom on the controllable carbon vacancies in graphene to realize different single atom catalyst is still highly desirable. The decorated metal atom is three-bond coordinated on SV graphene, while it is four-bond coordinated on DV graphene, indicating that the chemical activity of the decorated metal atom on different carbon vacancies should be different due to the different chemical environment. Furthermore, as mentioned above divacancy is commonly present in graphene obtained through chemical synthesis. However, only the catalytic activity of Fe-decorated DV graphene has been studied (Tang et al., 2016; Liu et al., 2017). Therefore, further research about the catalytic performance of DV graphene decorated with metal atom for CO oxidation is needed.

For the CO oxidation, Langmuir-Hinshelwood (LH) and Eley-Rideal (ER) are two mainly mechanisms. Along the ER mechanism, the activated O_2_ molecule directly reacts with the free CO molecule, where the activation of O_2_ is the rate-limiting step (Lu et al., 2009). Alone the LH mechanism, the CO and O_2_ molecules are first coadsorbed, and then react to form OOCO intermediate, which is the rate-limiting step for the oxidation progress (Lu et al., 2009). In general, the reaction energy barriers are proportional to the adsorption energy of adsorbed molecules on supported catalyst (Gong et al., 2004), indicating that the adsorption energy of adsorbed CO and O_2_ molecules could be a benchmark for the catalytic performance of graphene for CO oxidation. In addition, a larger adsorption energy for O_2_ molecule than that of CO is desired during the CO oxidation progress, because the preferential adsorption of CO will block the active sites and prevent the continuous oxidation reaction (Jiang et al., 2014; Tang et al., 2016). Therefore, the adsorption energy for O_2_ and CO on transition metals doped graphene is first calculated and the alternative decorating atom is chosen based on this rule.

In this work, by using first principles calculations, we will systematically study the CO oxidation mechanism on DV graphene decorated with transition metals from Sc to Zn, which are non-noble metals and commonly used to decorate two dimensional materials. The reaction barriers for each step are analyzed and the corresponding reaction mechanisms are discussed through analyzing the electronic property of the graphene systems.

### Calculation methods

The density functional theory (DFT) calculations are carried out by using Dmol^3^ package (Delley, 2000). Exchange-correlation functions are taken as generalized gradient approximation (GGA) with Perdew-Burke-Ernzerhof (PBE) (Perdew et al., 1996). The selection of exchange–correlation functional has evidential effect on the result of adsorption energies, while has much smaller effect on the reaction energy barriers (Roldán et al., 2009). DFT semicore pseudopotentials (DSPPs) core treatment is implemented for relativistic effects. Double numerical plus polarization (DNP) is employed as the basis set. The convergence tolerance of energy of 10^−5^ Hartree is taken (1 Hartree = 27.21 eV), and the maximal allowed force and displacement are 0.002 Hartree/Å and 0.005 Å, respectively. Linear synchronous transit/quadratic synchronous transit (LST/QST) (Halgren and Lipscomb, 1977) and nudged elastic band (NEB) (Henkelman and Jonsson, 2000) tools in Dmol^3^ module are used to investigate the minimum energy pathway for CO oxidation on graphene, which have been well validated to determine the transition state. Three-dimensional periodic boundary conditions are taken in the simulation. The simulation cell consists of a 4 × 4 graphene supercell with a vacuum width of 20 Å above the graphene layer to minimize the interlayer interaction. The *k*-point is set to 5 × 5 × 1, and all atoms are allowed to relax according to previous reports (Jiang et al., 2014). After structure relaxations, the density of states (DOS) are calculated with a finer *k*-point grid of 15 × 15 × 1. The DFT+D method within the Grimme scheme (Grimme, 2006) is used in all calculations to consider the van der Waals forces. The electron orbits of the free and adsorbed molecules are calculated with CASTEP code (Segall et al., 2002), where the ultrasoft pseudopotentials, GGA-PBE functional, an energy cutoff of 340 eV and 5 × 5 × 1 *k*-point meshes are used. We have compared the total energy of the graphene system with different spin state, and choose the proper spin state for the graphene system with the smallest total energy.

The adsorption energy *E*_ad_ of molecules on graphene is determined by,

(1) $$E_{\text{ad}}=E_{\text{molecules/graphene}}-\left(E_{\text{graphene}}+E_{\text{molecules}}\right)$$

where *E*_molecules/graphene_, *E*_graphene_, and *E*_molecule_ are total energies of the adsorbed graphene system, the isolate graphene and molecules respectively.

## Results and discussion

### Adsorption of O_2_ and CO on TM-DG

**Figure 1:**
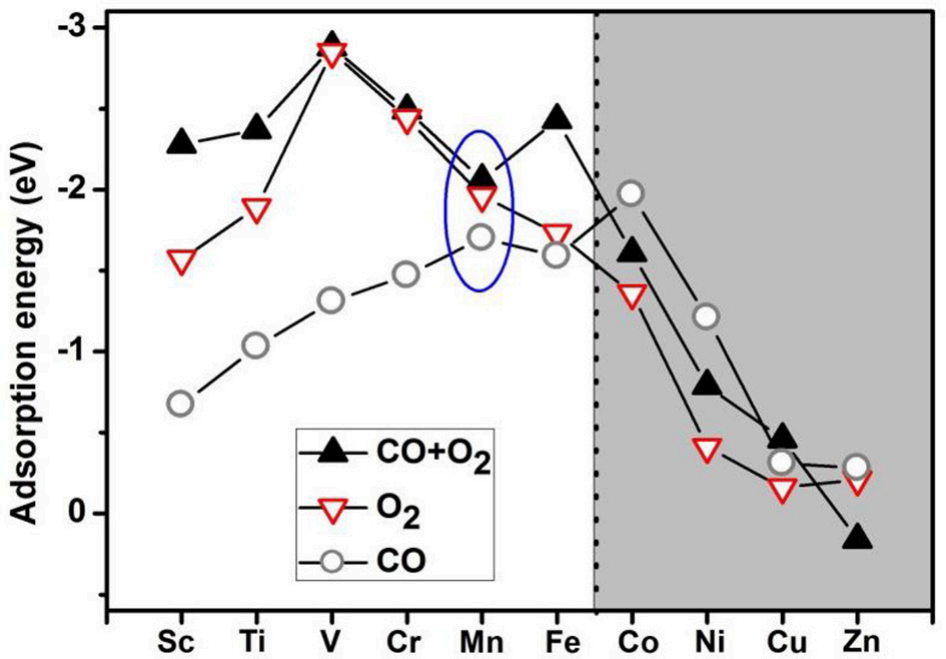
The adsorption energies of O_2_ and CO as well as the co-adsorption energy of O_2_ and CO on TM-DG, where the light and dark areas indicate the preferential adsorption of O_2_ and CO molecules, respectively.

Based on the literature results in the introduction section, it is expected that transition-metal-embedded divacancy graphene (TM-DG) could also exhibit excellent catalytic behaviors for CO oxidation, similar to the cases of graphene with single vacancy systems. Herein, we present systematic DFT calculations on the adsorption energy of O_2_ and CO molecules on the divacancy graphene embedded with transition metal (from Sc to Zn). To comprehensively understanding the adsorption behaviors of O_2_ and CO molecules on transition metal decorated divacancy graphene, the adsorption energies of O_2_ and CO, as well as the co-adsorption energy of O_2_ and CO molecules on TM-DG are shown in Figure 1, where we can see that the adsorption energy of O_2_ molecule is larger than that of CO molecule on TM-DG (from Sc to Fe), while the adsorption energy of CO molecule is larger than that of O_2_ molecule on TM-DG (from Co to Zn). This result indicates that the O_2_ molecule has priority during the adsorption progress and avoids the CO poisoning at the active sites on TM-DG (from Sc to Fe) during the CO oxidation. In addition, the co-adsorption energy of O_2_ and CO molecules on TM-DG (from Sc to Fe) is also shown in Figure 1, where a local adsorption energy minimum on Mn-DG is found, which indicates that Mn-DG can facilitate the CO oxidation better, due to the fact that the energy barrier is proportional to the adsorption energy of molecules (Gong et al., 2004). Therefore, CO oxidation on Mn-DG is mainly studied in the following, and the CO oxidation on other TM-DG (Sc, Ti, V, Cr and Fe) is also studied for comparison purpose.

### Electronic properties and stability of Mn-DG

**Figure 2:**
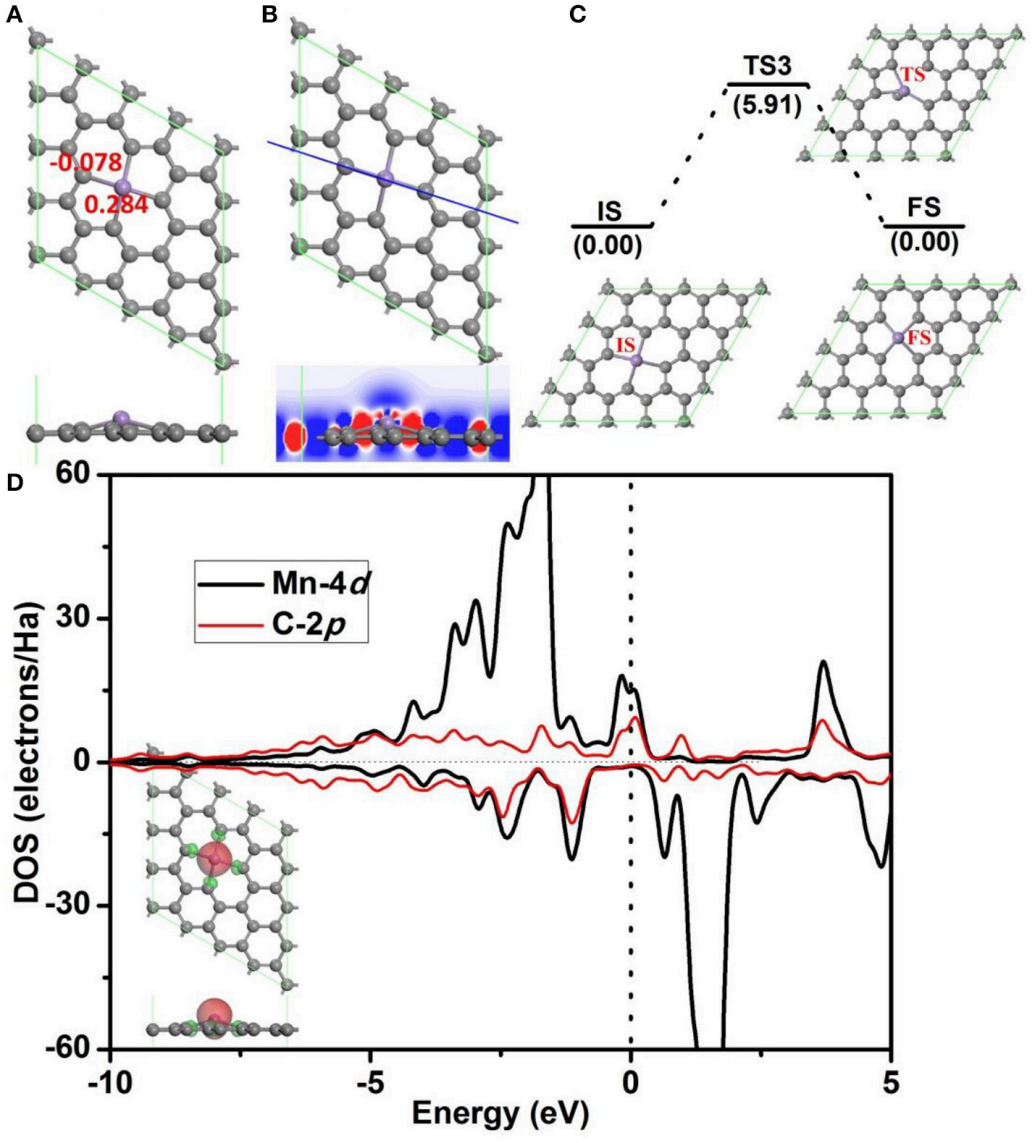
**(A)** The atomic structure of divacancy graphene embedded with Mn atom (Mn-DG), where the gray and pink atoms are C and Mn atoms, respectively, in this and following figures. The atomic charge obtained by Hirshfeld analysis near the Mn dopant is also given. **(B)** The differential charge density along C-Mn-C bonds for Mn-DG is shown, where the blue and red isosurfaces correspond to the increase in the number of electrons and the depletion zone, respectively. **(C)** The diffusion pathway of the Mn atom on Mn-DG, where IS, TS, and FS represent initial, transition and final structures, respectively, in this and following figures. **(D)** PDOS of Mn atom and C atom on the Mn-DG, where the vertical line indicates the Fermi level. Inset is the positive and negative spin density of Mn-DG shown in green and red, respectively.

Before further study the catalytic properties of Mn-DG, the electronic properties and structure stability of Mn-DG are first studied in Figure 2. After embedding Mn atom into the divancancy graphene through substituting two carbon atoms, the reconstructed structure of graphene is shown in Figure 2A, where four chemical Mn-C bonds are formed in graphene with bond length *l*_Mn−C_ = 1.99 Å. Mn atom is out of the plane of graphene with a distance of 0.68 Å due to the larger atomic radius of 1.79 Å compared with that of C 0.91 Å. The adsorption energy of the Mn atom on the divacancy graphene is −6.81 eV. The Hirshfeld charge distributions near the dopant are also given in Figure 2A, where the electron-deficiency position is formed for the Mn atom with losing electrons of 0.284 *e*, which promotes the adsorption of O_2_ and CO molecules. The differential charge density along C-Mn-C bonds for Mn-DG is further studied and is shown in Figure 2B, where the blue and red isosurfaces correspond to the increase in the number of electrons and the depletion zone, respectively. It shows that electrons accumulate near the doped Mn atom, indicating the high chemical active area.

The aggregation problems for the adsorbed metal atoms on substrate are significant for the catalytic performance, especially when the concentration of metal atom is high (Ao and Peeters, 2010a). To determine the possibility of aggregation for Mn atoms on divacancy graphene, the diffusion pathway of Mn atom to its neighboring positions is investigated based on DFT calculations (see Figure 2C), where the corresponding diffusion energy barrier for the decorated Mn atom is 5.91 eV. It is claimed that a surface reaction will occur when the reaction barrier is smaller than the critical value of *E*_cbar_ = 0.91 eV (Young, 2001), the decorated Mn atom on divacancy graphene is thus stable. In addition, the adsorption energy of Mn atom on divacancy graphene is −6.81 eV and it is much larger than the cohesive energy −2.92 eV/atom for Mn element (http://www.knowledgedoor.com/). Therefore, the Mn decorated divacancy graphene is quite stable without aggregation problems. Partial density of states (PDOS) are further analyzed to confirm the enhanced interactions between the Mn atom and graphene (Figure 2D), where the energy bands between the decorated Mn atom and carbon atoms overlap significantly. The electron-deficiency character is confirmed by the Fermi level crossing the valence band, and it is consistent with the Hirshfeld charge distributions. As shown in the insert of Figure 2D, the positive and negative spin density of Mn-DG is shown in green and red, respectively. Therefore, the magnetic moment of Mn-DG is 3.053 μ_B_, which is mainly contributed by the Mn atom. The remaining unsaturated *d* orbital of Mn atom is reactive, which can adsorb small molecules and promote the subsequent reactions.

### Adsorption of molecules on Mn-DG

**Figure 3:**
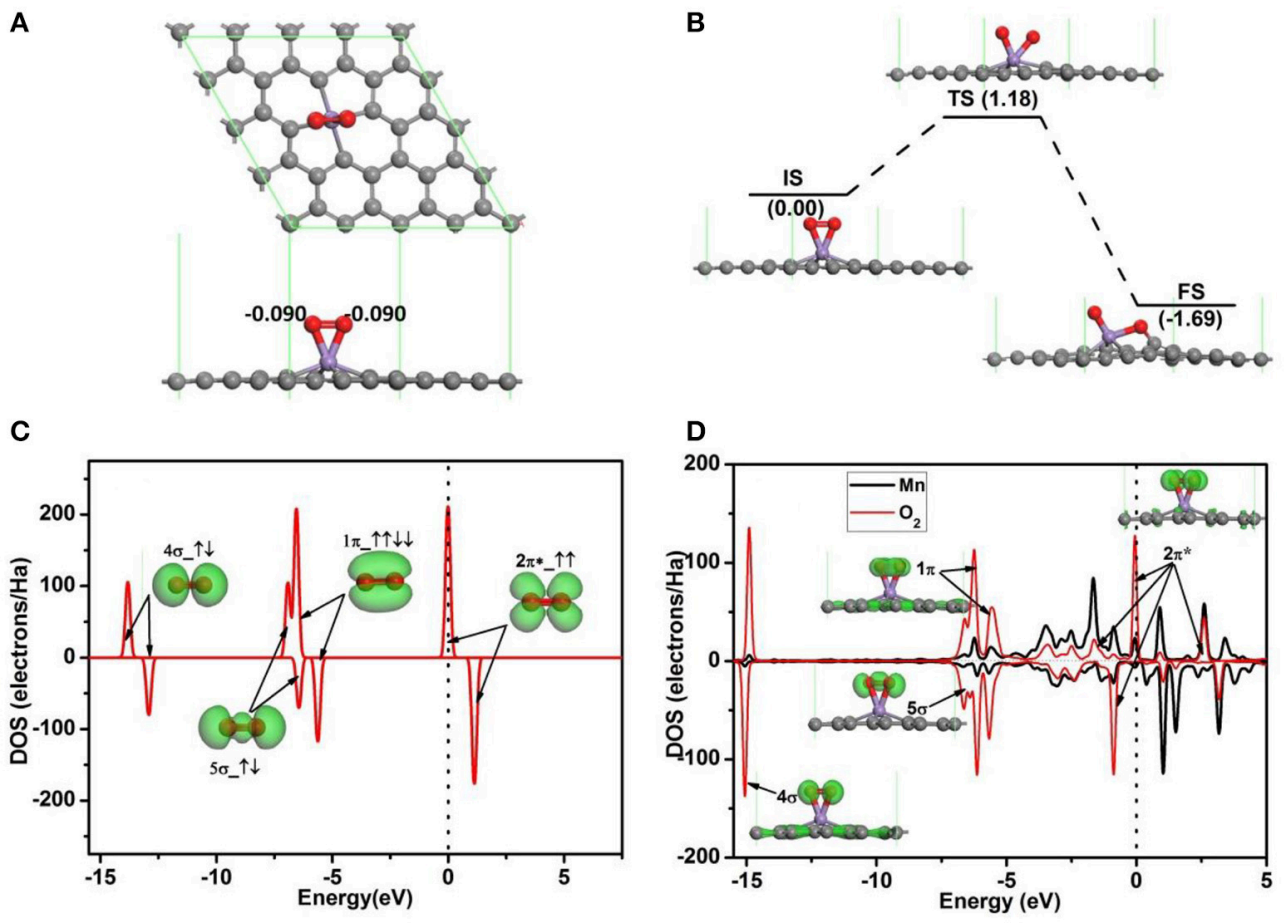
**(A)** The most stable structure of O_2_ adsorbed on Mn-DG. **(B)** The reaction pathway for the dissociative adsorption of O_2_ molecule on Mn-DG. **(C)** PDOS and orbitals of the free O_2_ molecule, where the number of electrons for each orbital is also shown by arrows. **(D)** PDOS of the adsorbed O_2_ molecule and Mn atom for Mn-DG, inset is the charge density of 4s, 5s, 1p, and 2p^*^ orbitals of the adsorbed O_2_ molecule. The vertical lines indicate the Fermi level.

To investigate the oxidation of CO on Mn-embedded divacancy graphene, the adsorptions of O_2_ and CO on Mn-DG are studied carefully. Figure 3A shows the most stable configuration for O_2_ molecule adsorbed on Mn-DG (*E*_ad_ = −1.96 eV), where the O-O bond is parallel to the graphene sheet, and ~0.18 *e* is transferred from Mn-DG to O_2_ molecule based on Hirshfeld charge analysis. To assess the stability of the adsorbed O_2_ molecule, the dissociative adsorption of O_2_ molecule on Mn-DG is then studied in Figure 3B. After NEB calculations, the dissociation reaction barrier for an O_2_ molecule on the Mn-DG is 1.18 eV > *E*_cbar_ = 0.91 eV (Young, 2001), which indicates that the adsorbed O_2_ molecule prefers to stay on Mn-DG in molecular state at room temperature. The PDOS of free O_2_ molecule is shown in Figure 3C, where the 2π^*^ anti-bond orbital is half filled (Honkala and Laasonen, 2000). Then, the PDOS of Mn atom and the adsorbed O_2_ molecule is shown in Figure 3D, where all orbitals of the adsorbed O_2_ molecule are also labeled to understand the interaction between the adsorbed O_2_ and Mn-DG. About 0.18 *e* is transferred from Mn-DG to the adsorbed O_2_ molecule based on Hirshfeld method, which occupies the O_2_-2π^*^ orbital above the Fermi level (see Figure 3C) and is confirmed by the new peaks for O_2_-2π^*^ orbital below the Fermi level (see Figure 3D). This charge transfer elongates the O-O bond from 1.23 Å in free O_2_ to 1.40 Å in adsorbed O_2_ molecule. The activated O_2_ molecule with longer O-O bond will be beneficial for the subsequent CO oxidation. The O_2_-2π^*^ orbitals and Mn atom is strong hybridized near the Fermi level (see Figure 3D), which mainly responses for the chemical adsorption of O_2_ molecule on Mn-DG.

**Figure 4:**
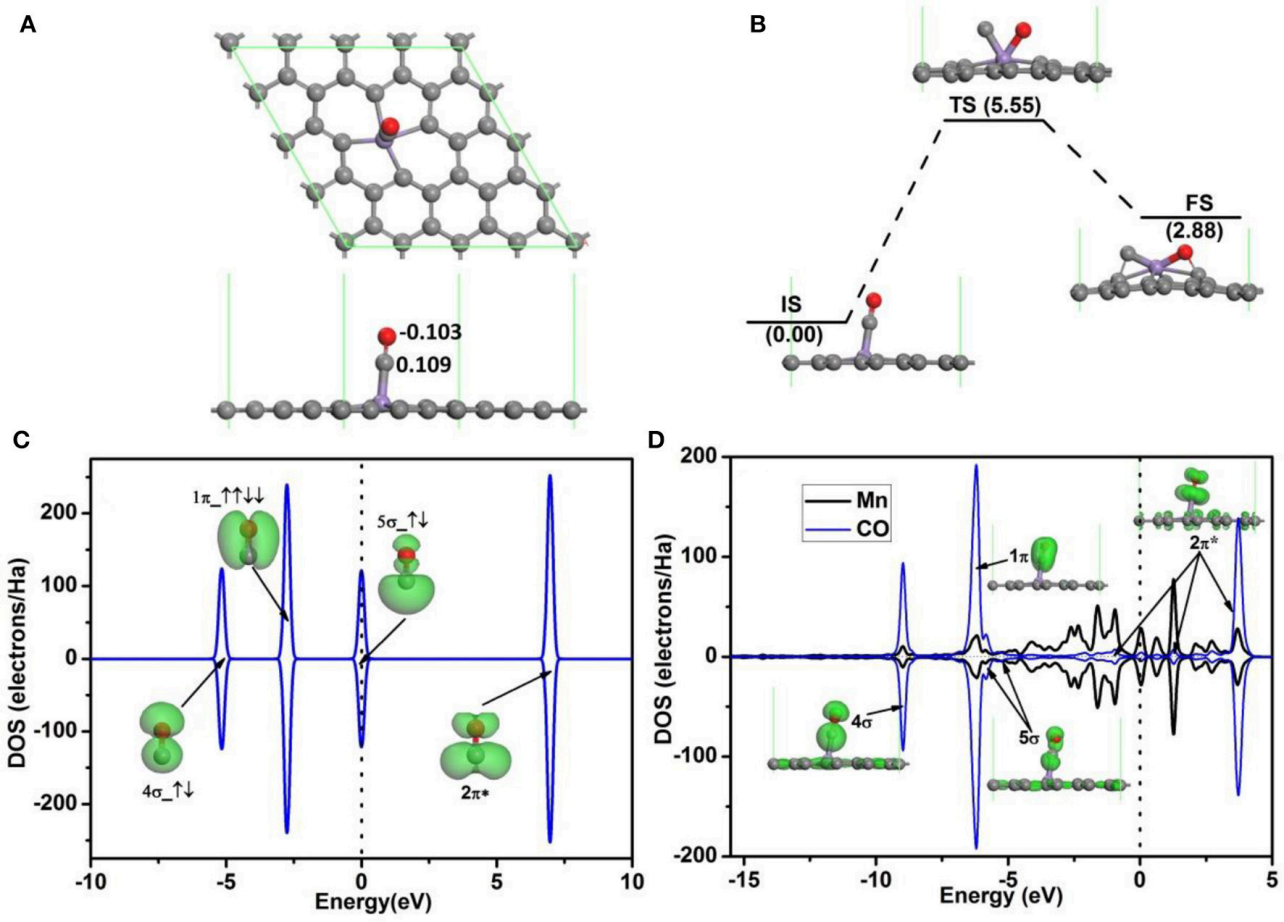
**(A)** The most stable structure of CO adsorbed on Mn-DG. **(B)** The reaction pathway for the dissociative adsorption of CO molecule on Mn-DG. **(C)** PDOS and orbitals of the free CO molecule, where the number of electrons for each orbital is also shown by arrows. **(D)** PDOS of the adsorbed CO molecule and Mn atom for the Mn-DG, inset is the charge density of 4σ, 1π, 5σ, and 2π^*^ orbitals of the adsorbed CO molecule. The vertical lines indicate the Fermi level.

The adsorption configuration of a CO molecule on Mn-DG is shown in Figure 4A, where the CO molecule is vertically adsorbed on the top of the decorated Mn atom. CO is chemically adsorbed on Mn-DG, which is confirmed by the chemical bond between Mn atom and the carbon atom of CO molecule. The adsorption energy of a CO molecule on Mn-DG is *E*_ad_ = −1.70 eV, and CO molecule loses 0.006 *e* to the Mn-DG. The binding energy *E*_b_ of C-O bond is 11.57 eV, which is much larger than *E*_b_ = 6.36 eV for O-O bond based on DFT calculations. Thus CO should be more difficult to be dissociated, which is confirmed by the fact that the dissociative energy barrier for CO molecule on Mn-DG is 5.55 eV based on DFT calculation as shown in Figure 4B. The PDOS of free CO molecule is shown in Figure 4C, where all orbitals are labeled. Figure 4D shows the PDOS of the adsorbed CO molecule on Mn-embedded divacancy graphene, where the orbitals of adsorbed CO molecule are labeled and displayed. The 5σ peak of the CO molecule adsorbed on Mn-DG is significantly depressed than the free CO molecule due to the charge transfer. Although the 2π^*^ anti-bond orbital far above Fermi level for free CO molecule is fully empty, Mn atom transfers some electrons to CO-2π^*^ orbital due to the fact that CO-2π^*^ is close to the Fermi level at absorbed state, which slightly elongates the C-O bond from 1.14 Å for free CO molecule to 1.16 Å for adsorbed CO molecule. In addition, CO molecules will act as a donor with the carbon atom near the graphene surface, dur to fact that the CO-5σ orbital locates on the carbon atom (Leenaerts et al., 2008). This agrees with the above Hirshfeld analysis result. The above discussions indicate that O_2_ and CO have strong interactions with Mn-DG (corresponding adsorption energies are −1.96 and −1.70 eV, respectively), but the adsorption of O_2_ is much stronger. The O_2_ molecule is activated on Mn-DG, which will facilitate the CO oxidation progress on graphene.

### CO oxidation on Mn-DG

Two reaction mechanisms have been established for the oxidation of CO molecule: Langmuir-Hinshelwood (LH) mechanism and Eley-Rideal (ER) mechanism (Molina and Hammer, 2005; An et al., 2008; Lu et al., 2009; Li et al., 2010; Song et al., 2011; Tang et al., 2012; Zhao et al., 2012; Jiang et al., 2014). For the ER mechanism, the O_2_ molecule is first adsorbed and activated by the Mn-DG, then a free CO molecule approaches the substrate to form an intermediate product. Along the LH mechanism, the O_2_ and CO molecules first co-adsorb on the Mn-DG, and then form an intermediate product. Since O_2_ has a larger adsorption energy (−1.96 eV) on Mn-DG than that of CO (−1.70 eV), the adsorption of O_2_ on Mn-DG has higher priority, thus the ER mechanism for the CO oxidation seems to be favorable. However, the lower co-adsorption energy for O_2_ and CO molecules (−2.06 eV) indicates that O_2_ and CO may co-adsorb on Mn atom as discussed in literatures (Lu et al., 2009; Song et al., 2011; Tang et al., 2012; Jiang et al., 2014). Therefore, both mechanisms for CO oxidation will be discussed in the following.

**Figure 5:**
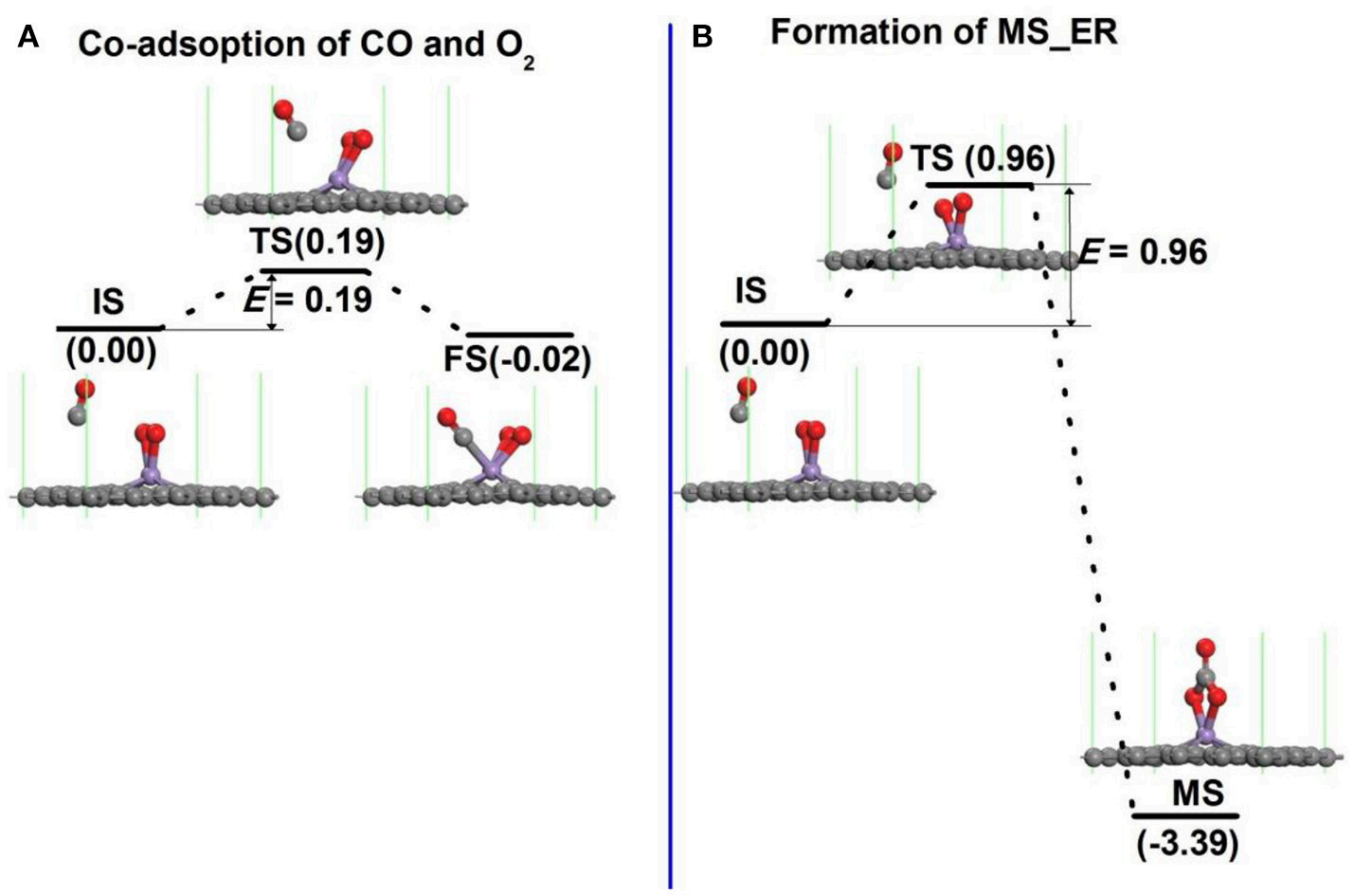
**(A)** The reaction pathway for the adsorption of CO molecule on Mn-embedded graphene with pre-adsorbed O_2_ molecule. **(B)** The primary reaction step for the oxidation of CO molecule on Mn-embedded graphene for ER mechanism.

In order to search the preferred reaction path for CO oxidation on Mn-DG, the first reaction step along both LH and ER mechanisms is studied in Figure 5. If the LH mechanism is more favorable, the free CO molecule will co-adsorb on Mn atom with pre-adsorbed O_2_ molecule after overcoming a small reaction barrier. If the ER mechanism is more favorable, the free CO molecule will react with the O_2_ molecule to form an intermediate (OOCO). The structure of physisorbed CO molecule on the Mn-DG with pre-adsorbed O_2_ molecule was selected as the IS after studying all adsorption configurations as shown in Figure 5A. The reaction profile for the co-adsorption of CO and O_2_ molecules on Mn atom is shown in Figure 5A, where the CO and O_2_ are titled toward the graphene surface at TS state. After overcoming an energy barrier of 0.19 eV, the CO and O_2_ co-adsorb on Mn-DG with releasing energy of 0.02 eV, as shown in Figure 5A.

Figure 5B shows the reaction profile for intermediate product along the ER mechanism. When the free CO approaches the Mn atom, the O-O bond is broken (see TS in Figure 5B). After overcoming a relative large energy barrier of 0.96 eV, the C atom from the CO binds with two O atoms from the dissociation of the O_2_ molecule to form a OOCO complex over the Mn atom (see FS in Figure 5B). This exothermic process releases an energy of 3.39 eV.

The effect of temperature on the energy barriers is considered. The free energy change (Δ*G*) between the reactant and transition state is considered as the temperature-dependent energy barrier *E*′_bar_ and Δ*G* = Δ*H*- TΔ*S*, where Δ*H* is the enthalpy change, Δ*S* is the entropy change, and *T* is the room temperature (298.15 K). In addition, Δ*H* = (Δ*U* + *P*Δ*V*), Δ*U* = (Δ*E*_tot_ + Δ*E*_vib_ + Δ*E*_trans_ + Δ*E*_rot_) and Δ*S* = Δ*S*_vib_ + Δ*S*_trans_ + Δ*S*_rot_, where Δ*U* is the internal energy change, Δ*E*_tot_ is the change of total electronic energy, the *vib, trans* and *rot* indicate vibration, translation and rotation, respectively, which can be obtained through calculations of vibrational frequency. The temperature-dependent energy barriers at 298.15 K in Figure 5 are: *E*′_bar_ = 0.33 eV in Figure 5A and *E*′_bar_ = 1.15 eV in Figure 5B. This indicates that the free CO molecule will desorb from substrate with increasing temperature, and the reaction is more difficult to occur. The co-adsorption of CO and O_2_ on Mn-DG in Figure 5A will happen at 298.15 K due to the lower reaction barrier than the critical barrier of *E*_cbar_ = 0.91 eV (Young, 2001), thus the LH mechanism is mainly studied for the CO oxidation in the following.

**Figure 6:**
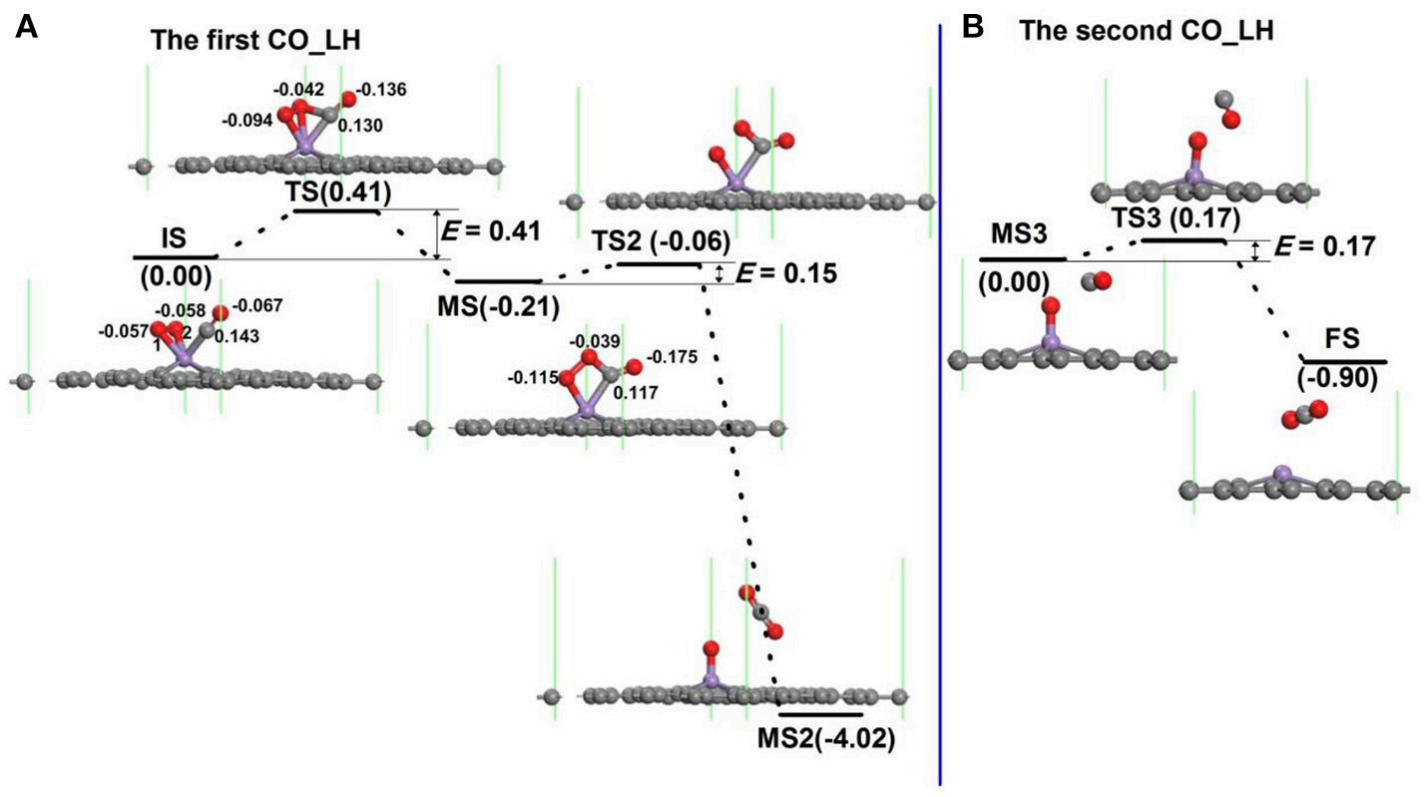
The reaction pathway of CO oxidation on Mn-embedded graphene for LH mechanism: production of the first CO_2_ molecule **(A)**, and the second CO_2_ moelcule **(B)**. The energy profile for the formation of CO_2_ molecule is also shown. The unit of *E* is eV, where *E* is the energy barrier. The Hirshfeld charge near the adsorbate is also shown.

For oxidation of CO on the Mn-DG along LH mechanism, several steps and also intermediate products (MS) for the oxidation procedure exist (Lu et al., 2009; Song et al., 2011; Tang et al., 2012; Jiang et al., 2014). For each step, e.g., from initial state to intermediate state in Figure 6, a transition state also exists. The configuration of co-adsorbed CO and O_2_ molecules on Mn-DG is taken as the reactant (IS in Figure 6A) based on the above discussions. At transition state, one O-Mn bond changes from 1.93 to 2.10 Å and a C-O bond between CO and O_2_ is formed. After overcoming an energy barrier of 0.41 eV, an OOCO intermediate (MS in Figure 6A) is formed and the elongated O-Mn bond is cleaved. Then the first CO_2_ molecule (MS2) is formed on Mn-DG after overcoming an energy barrier of 0.15 eV (see Figure 6A), where the CO_2_ is physically adsorbed on Mn-DG and its adsorption energy is −0.20 eV. The reaction for this step can release a heat of 3.81 eV, which can sufficiently overcome the adsorption energy of CO_2_, and the first produced CO_2_ molecule would desorb from the Mn-DG efficiently. The O-O bond for OOCO configuration in MS is broken at TS2, and a CO_2_ molecule with bond angle of 126.6° is formed. Figure 6B shows the formation of the second CO_2_ molecule on Mn-embedded divacancy graphene. The subsequent CO molecule will react with the remaining O atom to produce CO_2_ after surmounting an energy barrier of 0.17 eV (see Figure 6B), similar to the Au-embedded graphene (Lu et al., 2009). The reaction for this step releases a heat of 0.90 eV, which can also surmount the adsorption energy of CO_2_ (−0.27 eV) in Mn-DG, and the second CO_2_ molecule will desorb from Mn-DG efficiently. The reaction profile for the production of the two CO_2_ molecules is shown in Figure 6, where the rate limiting step is the formation of OOCO intermediate and the energy barrier of 0.41 eV is quite small. Therefore, the CO oxidation reaction along the LH mechanism releases 0.21 eV, 3.81 eV, and 0.90 eV for step 1, step 2, and step 3, respectively, which indicates the favorable thermodynamics for the CO oxidation on Mn-DG. In addition, the small energy barrier of 0.41 eV for the rate limiting step indicates the favorable kinetics for the CO oxidation on Mn-DG.

The energy barrier of each step for the CO oxidation along LH mechanism on divacancy graphene decorated with transition metals (from Sc to Fe) are also studied as shown in Table 1, where the rate limiting energy barriers are 1.15, 0.95, 0.85, 1.17, 0.41, and 0.88 eV for Sc-, Ti-, V-, Cr-, Mn-, and Fe-DG, respectively. The reaction barrier is the smallest on Mn-DG, which confirms the fact that the reaction barriers of molecules are proportional to the adsorption energy on supported catalyst (Gong et al., 2004), and the co-adsorption energy of adsorbed CO and O_2_ molecules can be a benchmark for the catalytic performance of the graphene for CO oxidation. Therefore, CO can be oxidized easily on Mn-DG at low temperature, indicating that Mn-DG is an excellent candidate during the catalysts for CO oxidation.

**Table 1:** The reaction energy barriers of each step for the CO oxidation on divacancy graphene decorated with transition metal (from Sc to Fe) along the LH mechanism, where *E*_bar1_, *E*_bar2_, and *E*_bar3_ are the energy barriers for step 1, step 2, and step 3 during CO oxidation, respectively.

	***E*_*bar*1_ (eV)**	***E*_*bar*2_ (eV)**	***E*_*bar*3_ (eV)**
Sc-DG	1.15	0.38	0.15
Ti-DG	0.95	0.24	0.43
V-DG	0.65	0.25	0.85
Cr-DG	1.17	0.22	1.10
Mn-DG	0.41	0.15	0.17
Fe-DG	0.88	0.13	0.06

The energy barriers at 298.15 K for each step of CO oxidation on Mn-DG in Figure 6 are: *E*′_bar_ = 0.49 eV for step 1, *E*′_bar_ = 0.10 eV for step 2, and *E*′_bar_ = 0.25 eV for step 3, where the energy barriers for step 1 and step 3 slightly increase than those at 0 K. The reaction time for each step in Figure 6 is calculated by Arrhenius equation (Pauling, 1988)

(2) $$\tau =\frac{1}{{}_{{}_{ve}}\text{​}\left(\frac{-E_{bar}}{K_{B}T}\right)}$$

where *K*_B_ is the Boltzmann constant, ν is in order of 10^12^ Hz, and *T* = 298.15 K. τ_1_ = 1.9 × 10^−4^ s for step 1, τ_2_ = 4.9 × 10^−11^ s for step 2, and τ_3_ = 1.7 × 10^−8^ s for step 3, respectively. Therefore, the CO oxidation on Mn-DG along LH mechanism has fast kinetics.

The possibility for the reversing reaction of CO oxidation, i.e., for the dissociation of the first and the second CO_2_ molecule into CO molecule and O atom on Mn-DG are further considered as shown in Figure 6. The reversing energy barriers for the step 1, step 2, and step 3 based on DFT calculations are 0.62, 3.96, and 1.07 eV, respectively. After considering the effect of temperature, the reversing energy barriers at 298.15 K are: *E*′_bar_ = 0.69 eV for step 1, *E*′_bar_ = 4.05 eV for step 2, *E*′_bar_ = 1.01 eV for step 3, while the corresponding reaction time is: τ_1_ = 0.44 s for step 1, τ_2_ = 2.3 × 10^56^ s for step 2, and τ_3_ = 1.1 × 10^5^ s for step 3. The long reaction time indicates that the reversing reactions for the CO oxidation on Mn-DG are hardly to occur and the CO oxidation can be finished thoroughly.

**Figure 7:**
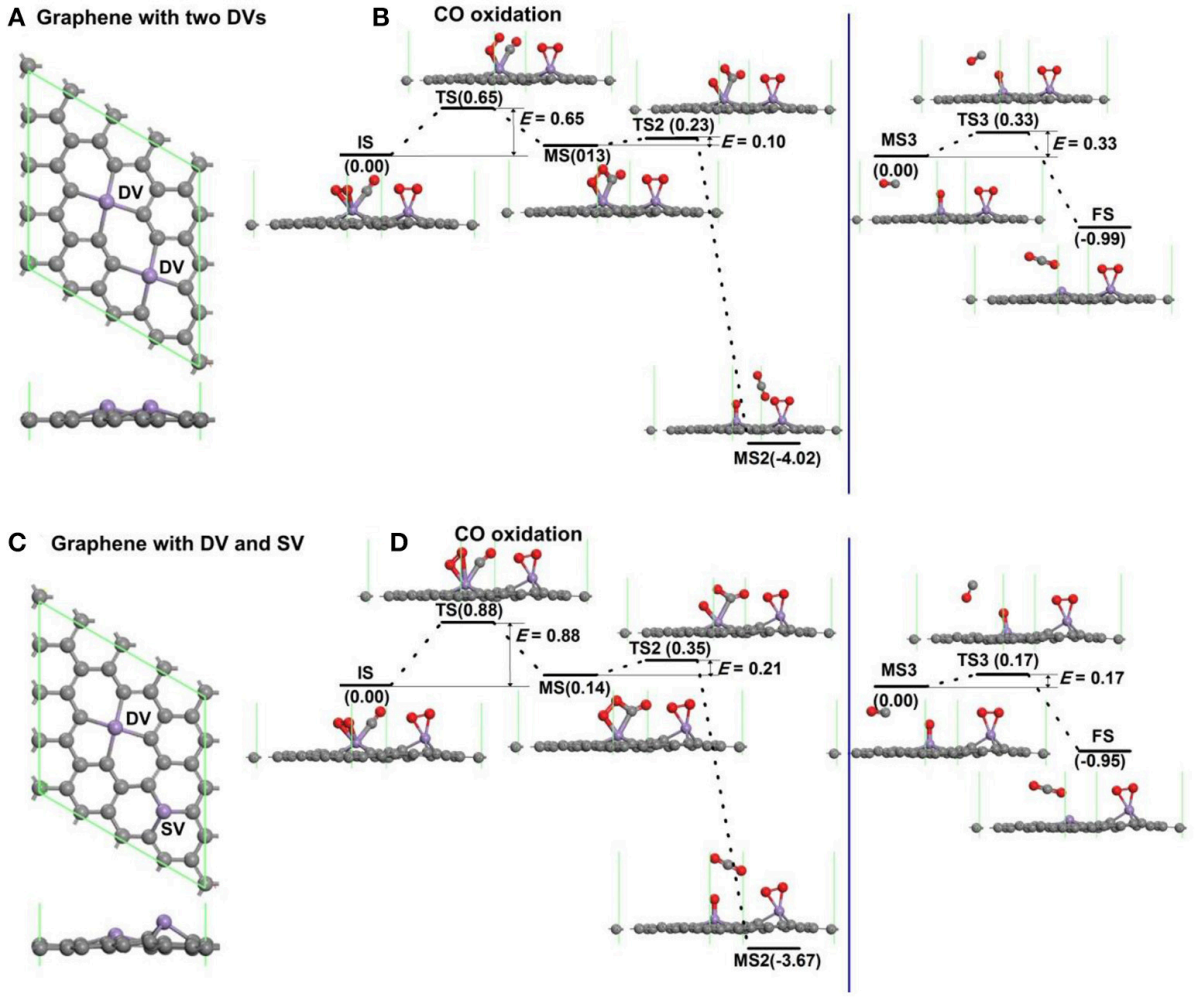
The atomic structure of graphene with two divacancies embedded with Mn atoms **(A)**, and the corresponding reaction pathway of CO oxidation along LH mechanism **(B)**. The atomic structure of graphene with one divacancy and one single vacancy embedded with Mn atoms **(C)**, and the corresponding reaction pathway of CO oxidation along LH mechanism **(D)**.

To consider the effect of interactions between vacancies on the oxidation of CO, the graphene with more vacancies in the 4 × 4 supercell is studied as shown in Figure 7. The atomic structure of graphene with two divacancies embedded with Mn atoms is shown in Figure 7A, and the corresponding reaction pathway of CO oxidation for LH mechanism is shown in Figure 7B, where the rate limiting energy barrier is 0.65 eV for the formation of OOCO complex. The atomic structure of graphene with one divacancy and one single vacancy embedded with Mn atoms is shown in Figure 7C, and the corresponding reaction pathway of CO oxidation for LH mechanism is shown in Figure 7D, where the rate limiting energy barrier is 0.88 eV for the formation of OOCO complex. Therefore, the reaction barriers on graphene with two double vacancies or one single and one double vacancies in the supercell in Figure 7 are both large than that on graphene with one double vacancy in Figure 6. Considering that the distances between the nearest Mn dopants in Figures 7A,**B** are 4.295 Å and 5.062 Å, while that in the periodic supercell with one double vacancy in Figure 2A is 9.908 Å with *E*_bar_ = 0.41 eV, thus the relative larger distance between carbon vacancies is beneficial for the CO oxidation on Mn-DG.

### Origin of the high activity of Mn-DG

**Figure 8:**
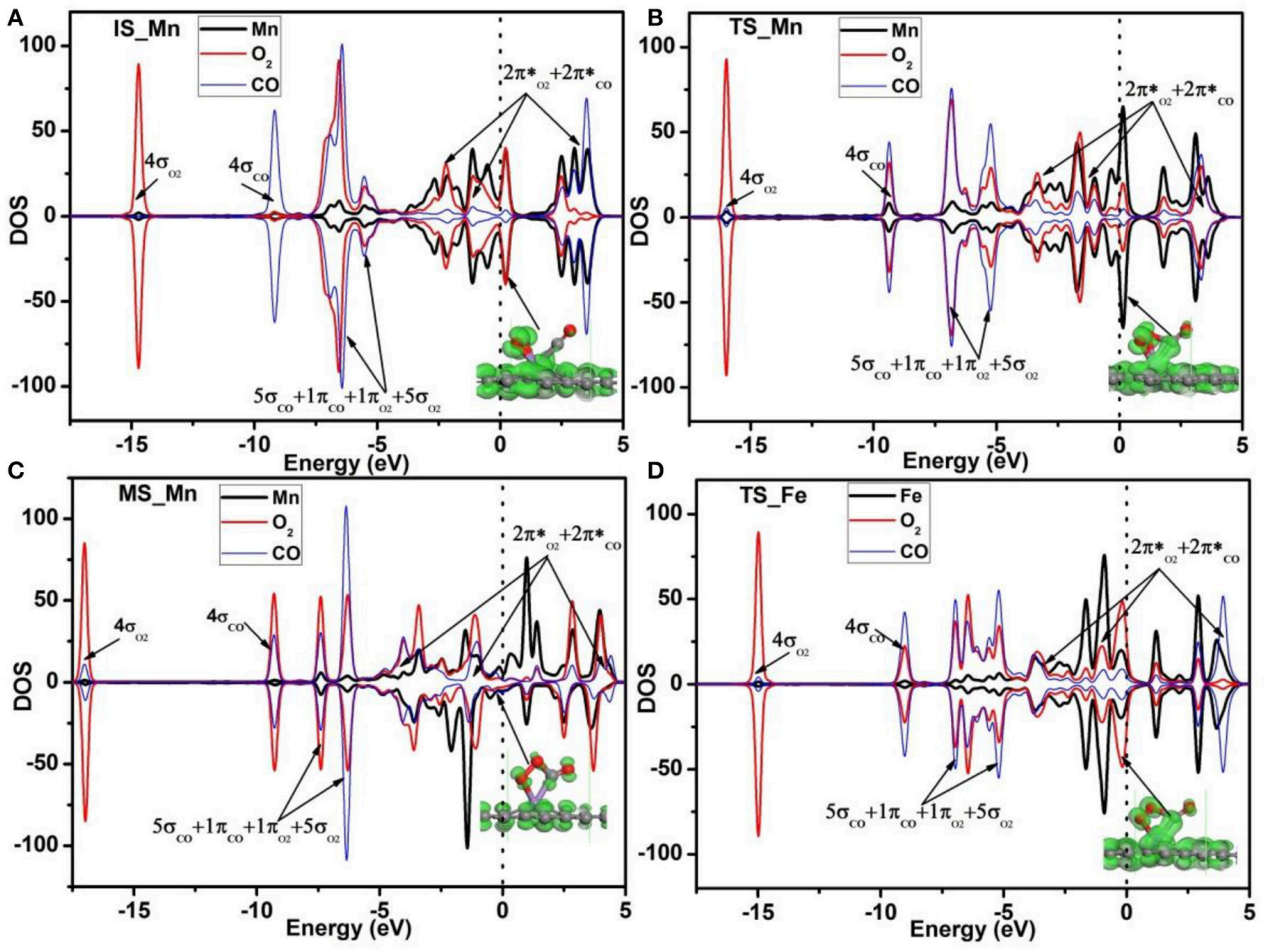
PDOS of adsorbed O_2_, CO, and Mn atom on Mn-DG for the IS **(A)**, TS **(B)**, and MS **(C)**. PDOS of adsorbed O_2_, CO and Fe atom on Fe-DG for the TS state is also shown in **(D)**. Red, blue and black curves represent PDOS of adsorbed O_2_, CO, and Mn (or Fe) atom, respectively. The orbitals of the adsorbed O_2_ and CO molecules are roughly labeled. Inset is the charge density of orbitals near the Fermi level. The vertical lines indicate the Fermi level.

To further understand the high activity of the Mn-DG for CO oxidation, the Hirshfeld charge near the adsorbed molecules along the LH reaction path is shown in Figure 6, and the corresponding PDOS near the adsorbed molecules is shown in Figure 8. O_2_ obtains 0.115 *e* while CO loses 0.076 *e* for the initial state in Figure 6A. The empty component of the O_2_-2π^*^ and CO-2π^*^ orbitals are partially filled (see Figure 8A) due to the electron transformation during the adsorption, which causes the elongation of the O-O bond and C-O bond to 1.34 Å and 1.15 Å, respectively. At transition state, the charge of adsorbed O_2_ is −0.136 *e* while that of adsorbed CO is −0.006 *e* in Figure 6A. This indicates that the O_2_-2π^*^ and CO-2π^*^ orbitals are more filled as shown in Figure 8B, which is confirmed by the elongation of the O-O bond and C-O bond to 1.46 and 1.17 Å, respectively. After surmounting the energy barrier, more charge transfers from graphene to the adsorbed O_2_ and CO, where the charge of O_2_ is −0.154 *e* while that of CO is −0.058 *e* for MS in Figure 6A. The O_2_-2p^*^ and CO-2π^*^ orbitals are more filled as shown in Figure 8C. Therefore, the Mn-DG can tune the charge distributions of the adsorbed O_2_ and CO, and the charge transfer from Mn-DG to O_2_ and CO molecules plays an important role for the OOCO intermediate formation.

Due to the fact that Fe is a commonly used dopant and the energy barrier for the first step on Fe-DG is 0.88 eV for CO oxidation, which is larger than that of 0.41 eV on Mn-DG, the PDOS of the TS configurations on Fe-DG is analyzed to further understood the mechanism for depressing the formation energy barrier of OOCO intermediate, as shown in Figure 8D. It can be seen that the overlapping area between O_2_-2π^*^ and Mn-3*d* orbitals at Fermi level on Mn-DG (see Figure 8B) becomes much weaker compared with that on Fe-DG (see Figure 8D), indicating that the interaction of Mn-O bond is significant weakened on Mn-DG. It is reported that weaker interaction nearby the Fermi level generally causes smaller reaction barrier (Arellano et al., 2000; Ao and Peeters, 2010b; Jiang et al., 2016). In addition, the interaction from −16 to −5 eV is strengthened on Mn-DG (see Figures 8B,D). Therefore, the enhanced interactions in the low energy range could lead to smaller reaction barrier for the CO oxidation on Mn-DG.

## Conclusion

The oxidation of CO molecule on transition metals decorated graphene with divacancy (DG) has been studied by using DFT calculations. We found that Mn-DG has the best performance for the CO oxidation, while the LH mechanism is preferred, where the rate limiting energy barrier is only 0.41 eV, indicating the efficient oxidation process. The charge transfer from Mn-DG to the O_2_-2p^*^ and CO-2π^*^ orbitals through the Mn atom along the LH mechanism plays a key role for depressing the energy barrier of the CO oxidation. The results indicate that Mn-DG can be a noble-metal-free and efficient catalyst for CO oxidation.

## Author contributions

QJ, JZ, and ZA designed the research and wrote the paper. QJ carried out the simulation. HJH, HYH, and YW entered the discussion. All authors commented on the manuscript.

### Conflict of interest statement

The authors declare that the research was conducted in the absence of any commercial or financial relationships that could be construed as a potential conflict of interest.
